# Experimental Evidence of 2,3,7,8-Tetrachlordibenzo-p-Dioxin (TCDD) Transgenerational Effects on Reproductive Health

**DOI:** 10.3390/ijms22169091

**Published:** 2021-08-23

**Authors:** Laura Gaspari, Françoise Paris, Nicolas Kalfa, Marie-Odile Soyer-Gobillard, Charles Sultan, Samir Hamamah

**Affiliations:** 1 Unité d’Endocrinologie-Gynécologie Pédiatrique, Service de Pédiatrie, CHU Montpellier, University of Montpellier, 34090 Montpellier, France; dr.lauragaspari@gmail.com (L.G.); f-paris@chu-montpellier.fr (F.P.); pr.charles.sultan@gmail.com (C.S.); 2 Centre de Référence Maladies Rares du Développement Génital, Constitutif Sud, CHU Montpellier, University of Montpellier, Hôpital Lapeyronie, 34090 Montpellier, France; n-kalfa@chu-montpellier.fr; 3 INSERM 1203, Développement Embryonnaire Fertilité Environnement, University of Montpellier, 34295 Montpellier, France; 4 Département de Chirurgie Viscérale et Urologique Pédiatrique, CHU Montpellier, University of Montpellier, Hôpital Lapeyronie, 34090 Montpellier, France; 5 Institut Debrest de Santé Publique IDESP, UMR INSERM, University of Montpellier, 34090 Montpellier, France; 6 CNRS, Sorbonne University, 75006 Paris, France; elido66@orange.fr; 7 Association Hhorages-France, 95270 Asnières-sur-Oise, France; 8 Département de Biologie de la Reproduction, Biologie de la Reproduction/DPI et CECOS, CHU Montpellier, University of Montpellier, 34090 Montpellier, France

**Keywords:** dioxin (TCDD), multigenerational transmission, endocrine disruptors (EDs), epigenetic, murine models

## Abstract

Previous studies have demonstrated that endocrine disruptors (EDs) can promote the transgenerational inheritance of disease susceptibility. Among the many existing EDs, 2,3,7,8-tetrachlordibenzo-p-dioxin (TCDD) affects reproductive health, including in humans, following direct occupational exposure or environmental disasters, for instance the Agent Orange sprayed during the Vietnam War. Conversely, few studies have focused on TCDD multigenerational and transgenerational effects on human reproductive health, despite the high amount of evidence in animal models of such effects on male and female reproductive health that mimic human reproductive system disorders. Importantly, these studies show that paternal ancestral TCDD exposure substantially contributes to pregnancy outcome and fetal health, although pregnancy outcome is considered tightly related to the woman’s health. In this work, we conducted a systematic review of the literature and a knowledge synthesis in order (i) to describe the findings obtained in rodent models concerning TCDD transgenerational effects on reproductive health and (ii) to discuss the epigenetic molecular alterations that might be involved in this process. As ancestral toxicant exposure cannot be changed in humans, identifying the crucial reproductive functions that are negatively affected by such exposure may help clinicians to preserve male and female fertility and to avoid adverse pregnancy outcomes.

## 1. Introduction

It has now been determined that some adult diseases are initiated during fetal and neonatal development. The resultant theory, known as the developmental origins of health and disease [1], has already modified medical interest on the potential role of fetal/neonatal exposure to harmful events/stimuli. Particularly, there is evidence that mammals are highly vulnerable to the damaging effects of endocrine disruptors (EDs), especially during periods of increased susceptibility (i.e., windows of susceptibility). For the male and female reproductive systems, this includes crucial events, such as fertilization and chromosomal sex determination, the fetal period up to infancy, and puberty and adolescence [2,3].

Moreover, a considerable and growing body of evidence indicates that chemicals and environmental toxicants might favor a transgenerational phenotype, which has important health consequences [4,5,6,7,8]. This underlying biological mechanism is called transgenerational epigenetic inheritance, a form of non-genetic inheritance that involves the transmission of an altered epigenome and its phenotypes through the germline across generations in the absence of the direct environmental exposure that caused the alteration [6,9,10]. The most studied epigenetic mechanisms involved in transgenerational inheritance are DNA methylation, histone modifications, methylation profile changes, and non-coding RNAs, including microRNA, chromatin structure, and RNA methylation [11].

A variety of EDs that can induce epigenetic alterations in the germline have been implicated in the transgenerational epigenetic inheritance of disease susceptibility [6,9,10]. Among the expanding list of EDs, 2,3,7,8-tetrachlordibenzo-p-dioxin (TCDD, or dioxin) is considered the most toxic chemical produced by humans [12] and has been selected as the “prototypical toxicant” for studying ED transgenerational effects on reproductive health.

TCDD is a ubiquitous contaminant introduced in the environment as an unwanted by-product of manufacturing (chlorine, paper, herbicides, fungicides and color metal production), combustion and waste incineration, car traffic, and cigarette smoking [13]. Forest fires and volcanic eruptions are the greatest natural sources of TCDD [14]. In addition, humans have been exposed to dioxin due to man-made disasters (e.g., the industrial explosion at Seveso in Italy and, particularly, the 19 million liters of Agent Orange contaminated with very high TCDD concentrations that were sprayed in South Vietnam, Laos, and Cambodia from 1962 to 1971) [15,16]. Both incidents resulted in severe and long-term effects of dioxin exposure in humans. Because of its solubility in lipids, high chemical stability and resistance to biodegradation, TCDD easily accumulates in human and animal tissues [13]. The TCDD half-life is estimated to be 7 to 11 years in humans, weeks to years in rodents, and 25 to 100 years in the environment [10,13,17].

TCDD has been associated with several negative reproductive health effects in humans who have been directly exposed to this ECD due to their work, such as Russian and New Zealand pesticide producers, or following environmental disasters, such as the Vietnam War, and Italian, Chinese and Taiwanese industrial accidents [18,19,20,21,22,23,24,25,26,27,28,29,30,31,32,33,34,35,36,37,38]. However, only a few studies have analyzed the multigenerational effects of TCDD on female and male reproductive health, and these studies reported a decreased sex ratio (male/female), alteration in pubertal timing, impaired fecundability and fertility in women, and reduced semen concentration, count, and motility in men [22,26,32,33,34,38,39,40,41,42]. Their findings could be compared to the effects of fetal exposure to diethylstilbestrol (DES), a potent estrogen compound used for miscarriage prevention until 1975 in the USA and later in Europe, with known multigenerational outcomes. While DES binds to estrogen receptors (ERs) or progesterone receptors (PRs), TCDD inhibits ER-mediated gene transcription by indirect mechanisms, e.g., by inducing competition between ER and the aryl hydrocarbon receptor (AhR, or dioxin receptor; protein: AHR, gene: *AHR*) for their common co-activator AhR nuclear translocator (ARNT) [43,44]. However, little overlap between the genetic networks activated by DES and TCDD was observed [44].

Our group and others have reported an increased risk of hypospadias and “idiopathic” partial androgen insensitivity syndrome in sons of DES daughters (i.e., women exposed prenatally to DES) [45,46,47,48,49]. Titus et al. observed delayed menstrual regularization, higher odds of irregular menstrual periods and amenorrhea, and increased risk of preterm delivery and fewer live births in women whose mothers were exposed to DES in utero (i.e., third generation) [50,51] as well as a higher rate of birth defects in DES grandchildren [52]. Very recently, we reported the first case of primary clear cell carcinoma of the cervix in an 8-year-old DES granddaughter, possible evidence of multigenerational DES effects [53].

Rodents are very useful for assessing the potential endocrine disrupting activity of chemicals and for providing data on the possible mechanisms of action and associated adverse effects. Therefore, the aim of this review is to provide a general overview on the transgenerational effects of TCDD exposure on reproductive health in rodents and to discuss the epigenetic mechanisms that might be implicated in this transgenerational epigenetic inheritance. A better understanding the transgenerational effects of TCDD on reproductive health may help to predict ED consequences for the next generations of humans.

## 2. Materials and Methods

A systematic review of the literature identified from the PubMed database was performed until June 2021 in accordance with the PRISMA guidelines. A comprehensive search using keywords [(“dioxin” OR “2,3,7,8-Tetrachlordibenzo-p-dioxin”) AND (“mouse model” OR “murine model” OR “mice” OR “rat”) AND (“fertility” OR “reproductive toxicity” OR “testicular toxicity” OR “sperm” OR “ovarian toxicity” OR “preterm birth” OR “pregnancy”) AND (“trangenerational” OR “multigenerational” OR “multiple generations” OR “three-generation” OR “epigenetic”)] was conducted, including all studies from database inception until June 2021. Reviews, studies that did not publish a full manuscript, and publications that were not in English were excluded.

## 3. Results

### 3.1. Articles Selection

A total of 19 articles were retrieved from the literature search. After title/abstract screening for matching inclusion/exclusion criteria, nine of these papers were then assessed for eligibility. The references of all of the computer-identified publications were searched for additional studies. Ultimately, 15 studies were included in the data analysis [10,54,55,56,57,58,59,60,61,62,63,64,65,66,67].

### 3.2. TCDD Transgenerational Effects on Female Reproductive Health

**Figure 1:**
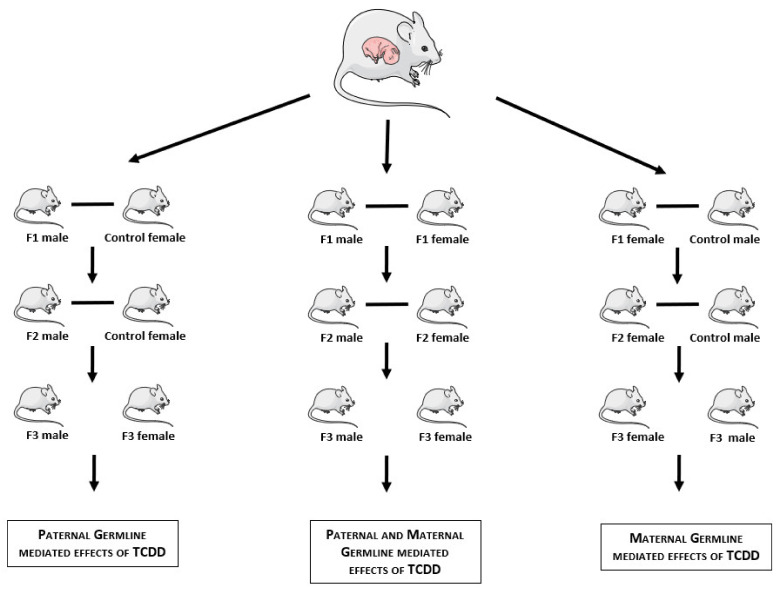
Summary of different experimental designs included in this systematic review [10,53,54,55,56,57,58,59,60,61,62,63,64,65,66].

Several studies provided evidence of transgenerational TCDD effects on female reproductive health that mimic the whole panel of human gynecological diseases, such as pubertal abnormality and menstrual disorders, endometriosis, polycystic ovarian syndrome (PCOS), subfertility, premature ovarian insufficiency (POI), and adverse pregnancy outcomes (Figure 1, Table 1). Endometriosis and PCOS have been largely analyzed in mammalian models of TCDD exposure because they have major roles in human fertility failure and are significant and independent risk factors of POI [68,69]. Endometriosis and adenomyosis are gynecological disorders that affect millions of women worldwide [70]. A possible effect of EDs with dioxin-like activity on endometriosis development has been suggested on the basis of the increased incidence and severity of spontaneous endometriosis cases in rhesus monkeys following dietary exposure to TCDD [71]. More recently, Bruner-Tran and Osteen’s group reported that in female C57BL/6 mice, in utero exposure to TCDD (10 μg/kg) by oral gavage at gestational day (GD) 15 results in an endometriosis-like uterine phenotype in adult life (F1 generation) [72]. Surprisingly, in the absence of any additional toxicant exposure, the three subsequent generations (F2–F4) presented adenomyosis, a frequently observed comorbidity in women with endometriosis [60]. Moreover, in the studied generations (F1–F3), the authors identified deep adenomyosis in only 25% of the mice with a history of successful pregnancy but in 100% of the infertile mice, suggesting a link between TCDD exposure, adenomyosis, and infertility. To identify the potential epigenetic consequences of developmental TCDD exposure, the authors analyzed uterine samples through methylation-specific PCR and found partial DNA methylation of the progesterone receptor (PR) followed by decreased endometrial progesterone sensitivity in 60% and 40% of the samples from directly in utero exposed F1 females and their F3 “granddaughters”, respectively [73]. Interestingly, the reduction in endometrial progesterone responsiveness is a well-recognized component of endometriosis [74,75].

Skinner and his group exposed pregnant Sprague Dawley rats (F0 generation) to TCDD (100 ng/kg/day) by intraperitoneal (IP) injection during the gonadal sex determination window (GDs: 8–14) and then monitored the appearance of ovarian alterations in adult F1 and F3 mice by histological analysis [55]. Ovarian follicle counting showed that the number of oocytes was significantly reduced in the ovaries of in utero exposed females (F1), which was also the case in the following generations. Specifically, the primordial follicle pool was decreased in approximately 60% and 35% of F1 and F3 ovaries, respectively [55], while the weight of the ovaries and uterus was unchanged until the F3 generation [54]. Importantly, the reduction of the primordial follicle pool size is a major physiological parameter in human POI [55]. In addition, the authors evaluated the presence of cystic structures (the third inclusion criterion for PCOS diagnosis) [76] in F1 and F3 ovaries. Interestingly, the number of small cysts was increased in ancestrally exposed F3 ovaries, but not in in utero exposed F1 ovaries, suggesting that this phenotype might be mainly explained by transgenerational epigenetic mechanisms and not by direct exposure to TCDD [55].

Zengli Yu and his group investigated the effect of exposing pregnant Sprague Dawley rats (F0) to TCDD (100 or 500 ng/kg/day, or 500 by gavage at GD 8–14) on follicle development and serum anti-Müllerian hormone (AMH) levels in the ovaries of F2 and F3 females [59]. The authors observed a significant decline in the number of primordial follicles in the F3 generation of the group exposed to the highest TCDD concentration [58] and also a reduction in the ovary weight and ovary organ coefficient (i.e., ovary weight/body weight) in the F3 generation compared to the controls (no exposure for F0 pregnant rats) [58]. However, there was no statistically significant difference in the serum AMH concentration between the F3 animals and the control group [59]. Interestingly, the apoptosis rate of the granulosa cells around the growing follicles was significantly increased in both F2 and F3 ovaries [59]. The elevated AMH level in the F2 ovaries, associated with the upregulation of the *Amh*/AMH receptor type II (*Amhr2*) mRNA expression, might inhibit progesterone secretion in granulosa cells, promoting their apoptosis and the subsequent arrest of follicular development [59]. The increased apoptosis rate of granulosa cells may directly or indirectly induce follicular atresia, thus increasing the risk of POI later in life [77].

Concerning TCDD effects on pubertal development and menstrual cycles, Yu’s group observed that in the progeny of Sprague Dawley rats exposed to TCDD, the vaginal opening, a physical hallmark of pubertal maturation, occurred significantly earlier, particularly in the group the was exposed to the highest TCDD dose in utero [58]. Similarly, Skinner’s group reported that 47% of ancestrally exposed F3 females presented early onset puberty, while in the never-exposed control group, only 6% of F3 females had pubertal abnormalities (mainly early puberty onset) [54,56]. In addition, Yu’s group observed a significantly increased rate of abnormal estrous cycles in the TCDD groups [58], as already reported in in utero exposed F1 rats by other authors [78].

Several studies have analyzed how TCDD exposure in pregnant rodents impairs the establishment and maintenance of pregnancy in the following generations (Figure 1, Table 1). Specifically, Bruner-Tran and Osteen’s group found that only 57% of ancestrally exposed F3 mice achieved pregnancy compared to 100% in the control (never exposed) group. Moreover, 25% of ancestrally exposed F3 fertile mice showed an increased risk of spontaneous preterm birth (PTB) (2/8 vs. 0/12 in the control F3 group) [57]. According to the authors, an epigenetic alteration leading to PR expression dysregulation might explain the reduced fertility and adverse pregnancy outcomes [57]. Over forty years ago, Murray et al. had already shown that in the F3 generation of Sprague Dawley rats in which both mating partners had ancestral TCDD exposure (0.1 μg TCDD/kg/day for 90 days before mating for the F0 progeny), the mean number of pups per litter at birth was significantly lower compared to the F3 controls (never exposed) [63], differently from what reported by Skinner and his team more recently [54].

Moreover, Bruner-Tran and Osteen analyzed the effect of latent mouse parvovirus (MPV) infection on the reproductive outcomes in mice where the F1 generation was exposed to TCDD in utero. This latent infection did not significantly affect the reproductive outcomes in the control F3 mice. Conversely, the infertility and PTB rates were higher in the MPV-infected mice than in the MPV-free ancestrally exposed F3 mice [57]. These data clearly demonstrate an important relationship between developmental TCDD exposure and lower resistance to an inflammatory challenge, mediated in this case by a latent chronic viral infection [57]. This higher sensitivity to inflammation, resulting from in utero exposure to TCDD, could also be transmitted through multiple generations [57]. The same group developed a model of ascending infection by group B streptococcus (GBS), an important PTB contributor in women, to investigate the impact of maternal vs. paternal developmental TCDD exposure on the response to this infection in adulthood [66]. They found that although gestation length was reduced in control mating pairs exposed to low-dose GBS, dams could fight the infection, and GBS transmission to pups was minimal [66]. In F1 mice with paternal or maternal developmental TCDD exposure history, GBS infection had different effects. Specifically, GBS infection in pregnant F1 females (mated to control male) resulted in 100% maternal and fetal mortality. Conversely, in control pregnant females mated to an exposed F1 male, GBS infection did not affect maternal health and gestation length but decreased neonatal survival compared to controls [66]. These studies clearly suggest a sex-dependent difference in placental development following early life TCDD exposure that affects the inflammatory response to infection at the maternal–fetal interface [66].

Concerning pregnancy outcomes, Bruner-Tran and Osteen’s group reported that the offspring of ancestrally exposed F3 males exhibited an intrauterine growth restriction (IUGR), and the placentae of these pregnancies were significantly smaller compared to control pregnancies [64]. Similarly, Sanabria et al. found that the body weight of the ancestrally exposed F3 dams tended to be lower than in the control group, although the sample size was small [62]. Finally, Rowlands et al. analyzed archived data from the study by Murray et al. and found no difference in the postnatal day 1 male/female sex ratio in any of the generations of exposed animals [65]. Similarly, no effect on sex ratio was reported in the ancestrally exposed F3 generation by Skinner and his group [54], which is different from what was observed by Ikeda et al. in ancestrally exposed F2 mice [79].

There is unquestionable evidence that puberty disorders and menstrual cycle abnormalities, endometriosis, PCOS, subfertility, and POI as well as adverse pregnancy outcomes are multifactorial, with a strong genetic predisposition modulated by the actual ED exposure [80,81,82,83]. However, experimental studies on TCDD effects clearly suggest that ancestral ED exposure through transgenerational epigenetic inheritance may also play a significant role in the pathophysiology of all female reproductive disorders, and this needs to be considered. Moreover, the transgenerational occurrence of subfertility and adverse pregnancy outcomes in control female partners highlights the role of toxicant-mediated epigenetic modifications in the male germline.

### 3.3. TCDD Transgenerational Effects on Male Reproduction

It is known that the male reproductive system is adversely affected by in utero or lactational exposure to TCDD in various animal species, including in rhesus monkeys, marmosets, guinea pigs, rats, mice, and chickens [84]. More recently, animal studies have focused on the transgenerational effects of TCDD on male reproduction (Figure 1, Table 1).

Gonad function was investigated in F3 mice by different authors. Skinner’s group exposed pregnant Sprague Dawley rats to TCDD (100 ng/kg/day) by IP injection during the gonadal sex determination window (GDs 8–14) [56]. In ancestrally exposed F3 males, testis, epididymis, and prostate weight as well as the incidence of testis and prostate diseases/abnormalities were not significantly different compared with never-exposed control animals [54]. Moreover, analysis of epididymal sperm concentration and motility percentage in ancestrally exposed F3 males did not suggest consistent transgenerational changes [56], as reported also by Sanabria et al. [62]. Similarly, Bruner-Tran and Osteen’s group showed that the mean sperm concentration was similar in ancestrally exposed F3 males and controls. However, they observed a significant reduction in morphologically normal sperm compared to controls (31% vs. 53%) [61], unlike Sanabria et al. [62]. Tail defects were the most common abnormality in all groups, while spermatocytes from ancestrally TCDD-exposed mice exhibited a slight increase in head, mid-piece, and acrosome defects compared to control mice [61]. The increased percentage of morphologically abnormal sperm suggests that TCDD affects germ cells (spermatotoxic ED) [62]. As altered macrophage numbers have been associated with poor testicular health, Bruner-Tran and Osteen’s group used an antibody against F4/80 to quantify the macrophages in the testes. They found that in ancestrally exposed F3 males, the number of resident macrophages was increased and that the sperm morphology was altered compared to controls (*p* < 0.001) and unexpectedly, also compared to F1 males (in utero exposed) [61]. Moreover, while the “baseline” chronic hyper-inflammatory profile in testes from the F1 and F3 animals was variable, the inflammatory response was increased in all animals with history of TCDD exposure following an exogenous challenge, resulting in reduced sperm quality and infertility [61].

Bruner-Tran and Osteen’s group analyzed the fertility and PTB rate when the F3 male mating partner was ancestrally exposed to TCDD [61]. As observed in ancestrally exposed F3 females, they found reduced pregnancy and PTB rates (50% and 35%, respectively) [61]. Similarly, Sanabria et al. investigated male fertility by intra-uterine insemination in the F3 generation from F1 males who had been exposed to TCDD in utero (0.1, 0.5, or 1.0 μg/kg at GD15). At GD9, the number of implanted embryos and of implanted embryos per corpus luteum was significantly lower in females fertilized by F3 males from the group ancestrally exposed to higher TCDD doses (0.5 or 1.0 μg/kg) [62].

Skinner’s group measured sex steroid hormone concentrations and found a drastic reduction in testosterone levels but no change in luteinizing hormone (LH) levels in ancestrally exposed F3 animals [56]. Bruner-Tran and Osteen’s group and Sanabria et al. reported a non-significant reduction in the serum level of testosterone [61,62], a well-known consequence of chronic inflammation in men [85]. In addition, they did not find any modification of the follicle-stimulating hormone (FSH) and LH levels, thus excluding an associated Sertoli cell dysfunction [61,62]. The percentages of testosterone reduction in these studies possibly differ because of differences in the experimental design. In the study by Skinner’s group, ancestrally exposed F3 males were from ancestrally exposed F2 parents. Conversely, in the studies by Bruner-Tran and Osteen and by Sanabria et al., in utero exposed F1 and then ancestrally exposed F2 males were mated with control females to obtain ancestrally exposed F3 males [56,61,62].

Finally, puberty onset investigation showed that in in utero exposed F1 animals, 40% of males had pubertal abnormalities, which mostly consisted of delayed pubertal onset [54]. Conversely, in ancestrally exposed F3 animals, only 5% of males had pubertal abnormalities (all delayed puberty) compared to 8% of control F3 males (mostly consisting of early pubertal onset) [54].

TCDD transgenerational effects on fertility have already been discussed in the previous chapter. In summary, Bruner-Tran and Osteen’s group reported reduced fertility and increased adverse pregnancy outcomes in ancestrally exposed F3 mice [61], and Sanabria et al. found a significant decrease in the number of implanted embryos and implanted embryos per corpus luteum when the male mating partner was ancestrally exposed to TCDD [62]. These findings provide clear evidence that ancestral exposure to TCDD during the critical stage of development results in transgenerational impaired reproductive health in adulthood, as reported for in utero exposed F1 animals [86].

Overall, the cited studies are of critical importance because their results suggest that ancestral fetal TCDD exposure adversely affects spermatogenesis and several important male reproductive functions, such as puberty onset, testosterone secretion, and fertility. Conversely, little is known about the underlying mechanisms. The next section discusses studies that tried to elucidate the possible transgenerational epigenetic mechanisms.

### 3.4. Transgenerational Epigenetic Mechanisms

Animal studies show that the effects of TCDD exposure on the female and male reproductive systems (from fetal life to puberty) are important because this ED can interfere with sex hormone signaling through interaction with steroid receptors or can modify the biosynthesis of the steroids required for sexual differentiation and later fertility and reproductive behavior [55,56,79,87,88]. In addition, TCDD has been reported to decrease antioxidant enzyme activity and increase the generation of reactive oxygen species (ROS) in female and male reproductive organs [89,90,91]. In a recent work, Lettieri et al. reported alterations in the protamines/histones ratio regarding the DNA binding of these proteins and their involvement in DNA oxidative damage in 84% of the young men living in dioxin-contaminated areas [92]. Interestingly, the same group evaluated a family case living in this area and reported a lower seminal antioxidant activity in the son than in the father [93].

The aryl hydrocarbon receptor (AhR, or dioxin receptor), a ligand-activated transcription factor, is the key factor that mediates TCDD outcomes. In its unbound state, AhR is located in the cytoplasm. Upon TCDD binding, it translocates into the nucleus, where it heterodimerizes with the AhR nuclear translocator (ARNT). The AhR/ARNT complex binds to the AhR response elements (AHRE, or XRE for xenobiotic responsive elements) in various target genes (i.e., cytochrome P450, family 1, subfamily A, polypeptide 1 *(Cyp1a1)*, and the aryl hydrocarbon receptor repressor *(Ahrr)*) to activate their expression, leading to harmful effects. In TCDD-exposed animals, the AhR transcriptional signaling pathway is primarily activated in the liver; however, AhR is also expressed in many cell types (i.e., pituitary, ovarian, testis, and germ cells) [88]. Particularly, it has been hypothesized that AhR activation modulates the signaling in the fetal pituitary gonadotropic cells, resulting in reduced expression of the FSH beta subunit and the LH beta subunit and consequently the decreased secretion of FSH and LH [88].

In male animal models, the suppression of steroidogenic gene expression (steroidogenic acute regulatory protein, cytochrome P450, family 11, subfamily a, polypeptide 1, cytochrome P450, family 17, subfamily a, polypeptide 1) in fetal and neonatal testes following TCDD exposure is partially caused by reduced pituitary LH production in the susceptibility window between GD 20 and postnatal day 4 [88]. Moreover, TCDD exposure reduces the expression of the cholesterol biosynthesis pathway genes in fetal testis, followed by decreased testosterone production [88]. Overall, decreased fetal FSH and testosterone levels following TCDD exposure might inhibit perinatal rat Sertoli cell proliferation and may ultimately reduce the spermatogenic output in adult rats [88].

In female animal models, TCDD exposure in utero significantly affects not only estradiol (E2), FSH, and AMH levels, but also results in impaired follicular development and premature ovarian failure [59,94]. This might be associated with the mRNA expression downregulation of the imprinted genes insulin-like growth factor 2 (*Igf2*) and *H19* and of the IGF2 protein [94,95] and the upregulation of *Amh* and *Amhr2* [59]. It has been suggested that altered *Igf2* and *H19* expression [94,95,96] could be explained by changes in DNA methylation. However, the mean DNA methylation was not changed at *Igf2* differentially methylated region 2 (DMR2) and at the *H19* imprinting control regions [94]. Recently, Devillers et al. reported the existence of an ovarian steroidogenesis window of vulnerability to AhR-mediated TCDD action during the prepubertal period, restricted to the late juvenile stage [97]. The authors administered TCDD to C57BL/6 mice at postnatal day 14 (infantile stage) or 28 (late juvenile stage). Quantification of circulating FSH and LH revealed no change. Conversely, in animals exposed at postnatal day 28, the relative ovarian expression of E2, *Cyp19a1*, *Cyp1a1*, the FSH receptor, and *Ahrr* was significantly increased [97]. This suggests the existence of a developmental period, the late juvenile stage (postnatal day 28), when TCDD acting via AhR could exert adverse effects on puberty onset and fertility by altering prepubertal ovarian function [97].

During fetal life, primordial germ cells undergo DNA demethylation during a sex specific program to generate sperm or eggs [98,99]. TCDD exposure during this critical window of vulnerability can alter germline epigenetic reprogramming. Sometimes, the altered DNA methylation appears to become permanent, and is propagated from the male and/or female germline to the zygote, resulting in an altered epigenome and transcriptome in the subsequent generations [10,11,98,100]. Transgenerational epigenetic inheritance describes the transmission of an altered epigenome and phenotype through the germline across generations in the absence of continuous direct environmental exposure [6,9,10]. The implicated epigenetic mechanisms are DNA methylation, histone modifications, non-coding RNAs, chromatin structure, and RNA methylation [10,11] (Figure 1, Table 2). These strongly intertwined processes determine whether a gene is expressed or silenced, playing a crucial role in cell and tissue development, through complex mechanisms that can affect transcript stability, DNA folding, nucleosome positioning, chromatin compaction, and nuclear organization [101].

In animal models, Skinner’s group reported that ancestral TCDD exposure could promote the transgenerational epigenetic inheritance of DNA methylation epimutations in sperm [54,55]. Specifically, they identified differentially methylated sites in sperm from ancestrally exposed animals compared to control F3 rats [56]. Using tiling arrays to analyze sperm samples from ancestrally exposed F3 rats, they detected 50 differentially DNA methylated regions in gene promoters, among which 28 were specific to TCDD exposure. Indeed, each ED has a unique signature of epigenetic alterations in F3 sperm [56]. Moreover, the authors identified DMR clusters, probably representing “epigenetic control regions”, where TCDD-specific DMRs may regulate gene activity (i.e., neuroblastoma ras oncogene, filaggrin, semaphorin 3B, Src homology 2 domain containing transforming protein 2, and heat shock protein 1) [56]. In addition to these ancestral epigenetic biomarkers in sperm, the same group identified a DNA sequence motif, termed “environmentally induced DNA methylation region 1” (EDM1) [100], that was associated with a high percentage of promoter regions in the TCDD exposure group [56]. More recently, the same group used the archived pathology slides and sperm samples from their previous studies [54,56] to identify ancestrally exposed F3 rats without (control) or with a single TCDD transgenerational pathology (i.e., late puberty, testis or prostate disease, obesity, kidney disease, tumor) [10]. They then analyzed frozen sperms samples from such animals by methylated-DNA immunoprecipitation sequencing (MeDIP-seq) to identify TCDD-induced sperm DMR epimutations. They observed a negligible overlap of the DMRs between groups (with/without disease) at the *p*-value (*p* < 1 × 10^−4^) threshold [10]. Moreover, they identified the genes associated with each disease-specific DMR dataset and that were linked to a specific disease [10]. For instance, in samples from ancestrally exposed F3 rats with testis disease, DMR-associated genes included genes linked to testis physiology and male infertility [10]. Similarly, Prokopec et al. analyzed the testes from ancestrally exposed F3 rats by targeted bisulfite sequencing of entire chromosomes or selected genes and detected many DMRs at specific genes, such as melanocortin 5 receptor (*Mc5r)*, protein phosphatase 1, regulatory subunit 27 *(Ppp1r27)*, family with sequence similarity 109, member a *(Fam109a)*, heat shock protein 8 *(Hspa8)*, peptidase inhibitor 16 *(Pi16)*, and RAS protein activator like-3 *(Rasal3)*. Conversely, they did not find any DMR in or around *Ahr* or any “AHR-core” genes [67]. The authors also showed that multiple olfactory receptors displayed patterns of differential DNA methylation, which is quite interesting because aversion to novel foodstuffs is a highly sensitive behavioral response to AHR agonists [67,102,103]. Moreover, the marked hyper-methylation of epidermal growth factor receptor found by Prokopec et al. in testis samples from ancestrally exposed F3 rats (62% vs. 0% in controls) is also of interest because it occurs within the gene body [67], a mechanism proposed to contribute to transgenerational plasticity in response to environmental stimuli [104] and that is linked to tumor development [105].

The male germ cells represent the paternal contribution to pregnancy (fetus but also placenta formation) [64]. Through microarray analysis, Bruner-Tran and Osteen’s group determined the epigenetic profile of placentae from pregnancies in which the father was (F1) or not in utero exposed to TCDD and identified 2171 DMRs, including progesterone receptor (*Pgr*) and *Igf2* [64]. Proper *Pgr* expression is critical for pregnancy maintenance, and placental *Igf2* plays an important role in regulating fetal growth [64]. Ding et al. obtained similar results in sperm from ancestrally exposed F3 males, suggesting that these epigenetic changes might be transmitted to the progeny [64]. Indeed, analysis of sperm and placenta samples revealed hypermethylation of *Pgr* and hypomethylation of *Igf2*, although only placental *Pgr* methylation changes were statistically significant (*p* < 0.05) [64]. Moreover, PGR and IGF2 mRNA and protein levels were reduced in placentae (pregnancies with ancestrally exposed F3 males), but only the decrease in *Pgr* was significantly different from controls [64]. In addition, the offspring of ancestrally exposed F3 males consistently exhibited IUGR, and the placentae of these pregnancies were significantly smaller than in control pregnancies [64]. Bruner-Tran and Osteen’s group also analyzed *H19* mRNA expression because this gene is in close proximity to *Igf2*, and these two genes have a reciprocal role in regulating fetal growth [64,94,106]. They found that *H19* expression was significantly reduced in ancestrally exposed F3 male-derived placentae [64]. In agreement, Wu et al. reported that in vitro exposure of pre-implantation mouse embryos to TCDD is associated with the repression of both *H19* and *Igf2* mRNA expression and with a significant reduction in fetal weight following embryo transfer to control dams [95]. Altogether, these data support the hypothesis that paternal exposure to TCDD leads to alterations in the placental epigenome that are associated with placental dysfunction, impaired fetal development, and decreased gestation length [64].

Conversely, only few studies have analyzed the transgenerational epigenetic inheritance mechanism(s) leading to female reproduction impairment. As AMH is mainly derived from follicular granulosa cells, Yu et al. quantified *Amh* and *Amhr2* mRNA levels in the ovaries [59] and found that *Amh* was upregulated in the ovaries of F1 and F2 and also F3 mice in the high-dose TCDD group, whereas *Amhr2* was only downregulated in F3 ovaries from the low-dose TCDD group compared to controls [59]. This suggests that in the F3 generation, the normal biological effects of AMH, which are primarily due to AMHR2 action, are reduced, leading to hormonal disorders and endocrine dysfunctions [59]. Accordingly, Peluso et al. reported that in women, altered AMH and AMHR2 mRNA expression can modify the biological activities of hormones, thus affecting follicle recruitment and development [107]. Besides the AMH/AMHR2 pathway, IGF2 and H19 have a role in folliculogenesis and oocyte development because they are secreted by oocytes and are implicated in granulosa cell proliferation in follicles and in steroidogenesis [58,108]. Yu et al. collected and purified ovaries from ancestrally exposed F3 rats to determine the transgenerational effects of TCDD exposure on IGF2 and H19 [58]. In agreement with a previous study on the transgenerational toxicity of TCDD in the liver [96], Yu et al. showed a trend towards *Igf2* downregulation and *H19* upregulation in the ovaries [58]. This suggests that ancestral TCDD exposure may transgenerationally impair adult ovary development and functions, possibly through the inhibition of the IGF2/H19 pathway [58].

## 4. Conclusions

ED transgenerational effects on reproductive health are not fully elucidated. Currently, the question of whether ED fetal exposure results in heritable epigenetic alterations that might negatively affect the reproductive function of future generations remains a key issue for researchers and clinicians.

This systematic review highlighted the occurrence of transgenerational effects, such as subfertility and adverse pregnancy outcomes, that might implicate TCDD-mediated epigenetic modifications in both germlines. The vast majority of EDs act in somatic cells during critical developmental windows to influence the development of a phenotype and/or disease that will not be inherited [9]. Conversely, if exposure to an ED (e.g., TCDD) occurs during the critical window of primordial germ cell development, the altered germ line phenotype may be transmitted to the subsequent generations [9].

It is important to stress that this systematic review is also associated with several limitations. In particular, the animal studies included in our analysis were heterogeneous in relation to animal species (rat, Sprague Dawley or Wistar; mouse, C57Bl/6), route of administration (oral gavage or IP injections), and TCDD timing and dose regimen used (0.1, 0.5 or 1.0, 10 μg/kg, single dose; 100 or 500 ng/kg/day during GD 8–14 or 0.001, 0.01 or 0.1 g/kg/day during 90 days prior to mating). In addition, many factors may contribute to differences between the results of controlled animal studies and the epidemiological findings in humans [101]. First, TCDD is usually found in the environment as a component of complex pollutant mixtures, a situation that is very different from the laboratory exposure conditions. Indeed, the doses used in experimental studies are higher than the estimated daily exposure to TCDD, and the experimental exposure routes are not comparable to daily human exposure. Nevertheless, the conclusions of experimental studies are relevant for humans because more and more data are becoming available on the health consequences of ancestral exposure to TCDD in the next generations. In addition, the findings on the effects of Orange Agent exposure during the Vietnam War as well as more recent exposure in Iraq and Afghanistan Veterans with deployment-related exposure to open-air burn pits are the major incentives for pursuing research on the transgenerational effects of TCDD.

As ancestral toxicant exposure cannot be modified, it is imperative to identify the reproductive processes potentially affected by such exposure in order to propose targeted therapies to preserve male and female fertility and to avoid adverse pregnancy outcomes. Furthermore, although pregnancy outcome is considered tightly related to a woman’s health, the father also substantially contributes to the pregnancy outcome and fetal health [64]. Therefore, clinicians must identify TCDD granddaughters and grandsons and offer them and their partners appropriate clinical follow-up.

## Figures and Tables

**Table 1:** TCDD transgenerational effects on the female and male reproductive health and pregnancy outcomes in rodent experimental models.

F3 Females	Paternal Germline	Paternal and Maternal Germline	Maternal Germline
Fertility			↓ [57] ^2^
Ovarian weight		N [54] ^1^	↓ [58] ^3^
Ovarian primordial follicles		↓ [55] ^1^	↓ [58] ^3^
Granulosa cell apoptosis around growing follicles			↑ [59] ^3^
AMH concentration			N [59] ^3^
Earlier pubertal maturation		↑ [54,56] ^1^	↑ [58] ^3^
Estrous cycle abnormalities			↑ [58] ^3^
Polycystic ovaries		↑ [54] ^1^	
Adenomyosis			↑ [60] ^2^
**F3 Males**	**Paternal Germline**	**Paternal and Maternal Germline**	**Maternal Germline**
Fertility	↓ [61] ^2^		
Testosterone concentration	↓ (ns) [61] ^2^ ↓ (ns) [62] ^4^	↓ [56] ^1^	
LH concentration	N [61] ^2^ N [62] ^4^	N [56] ^1^	
Sperm number	N [61] ^2^		
Normal sperm morphology	↓ [61] ^2^ N [62] ^4^		
Epididymal sperm concentration and motility	N [62] ^4^	N [56] ^1^	
Epididymis and prostate weight		N [54] ^1^	
Testis and prostate diseases/abnormalities		N [54] ^1^	
Pubertal development		N [54] ^1^	
**F3 Pregnancies**	**Paternal Germline**	**Paternal and Maternal Germline**	**Maternal Germline**
Premature birth	↑ [61] ^2^		↑ [57] ^2^
Litter size at birth		↓ [63] ^6^ N [54] ^1^	
Number of implanted embryos and number of implanted embryos per corpus luteum	↓ [62] ^4^		
Placental weight	↓ [64] ^5^		
Pup weight	↓ [64] ^5^		
Gestation survival index		↓ [63] ^6^	
Male/female sex ratio		N [65] ^6^ N [54] ^1^	
Ascending GBS infection during pregnancy	Maternal health and gestation length N, neonatal survival ↓ [66] ^5^		100% maternal and fetal mortality [66] ^5^
Infertility and premature birth after latent MPV			↑ [57] ^2^

Legend: N = normal value; AMH = anti-Müllerian hormone; ns = not significant; LH = luteinizing hormone; GBS = group B Streptococcus infection; MPV = mouse parvovirus infection; GD = gestation day.; ^1^—Rat, Sprague Dawley; TCDD 100 ng/kg/day IP injection in DMSO, GD 8–14 [54,56]; ^2^—Mouse, C57Bl/6; TCDD 10 μg/kg, single dose, oral gavage in corn oil, GD 15.5 [57]; ^3^—Rat, Sprague Dawley; TCDD 100 or 500 ng/kg/day, oral gavage in corn oil, GD 8–14 [58,59]; ^4^—Rat, Wistar; TCDD 0.1, 0.5 or 1.0 μg/kg, single dose, oral gavage in corn oil, GD15 [62]; ^5^—Mouse, C57Bl/6; TCDD 10 μg/kg, single dose, oral gavage in corn oil, GD 15.5 [64,66]; ^6^—Rat, Sprague Dawley; TCDD 0.001, 0.01 or 0.1 g/kg/day, in diet, 90 days prior to mating [63,65].

**Table 2:** Epigenetic mechanisms involved in TCDD transgenerational effects on female and male reproductive health in rodent models.

F3 Females	Paternal Germline	Paternal and Maternal Germline	Maternal Germline
			AMH mRNA ↓ and AMHR2 mRNA ↑ in the ovary [58] ^1^
			*Igf2* mRNA ↓ and *H19* mRNA ↑ in the ovary [66] ^1^
**F3 Males**	**Paternal Germline**	**Paternal and Maternal Germline**	**Maternal Germline**
	DMR epimutations in testes [67] ^2^	DMR epimutations in sperm [54] ^4^	
	DMR epimutations in sperm [64] ^3^	Disease-specific DMR epimutations in sperm [10] ^4^	
	DMR epimutations in male-derived placentae [64] ^3^		
	*Pgr*, *Igf2* and *H19* mRNA ↓ in male-derived placentae [64] ^3^		

Legend: DMR = DNA methylated regions; *Pgr* = progesterone receptor; *Igf2* = insulin-like growth factor 2; AMH = anti-Müllerian hormone; AMHR2 = anti-Müllerian hormone receptor type II; ^1^—Rat, Sprague Dawley; TCDD 100 or 500 ng/kg/day, oral gavage in corn oil, GD 8–14 [66]; ^2^—Rat, Sprague Dawley; TCDD 0, 30, 100, 300 or 1000 ng/kg, single dose, oral gavage in corn oil, GD 11 [67]; ^3^—Mouse, C57Bl/6; TCDD 10 μg/kg, single dose, oral gavage in corn oil, GD 15.5 [64]; ^4^—Rat, Sprague Dawley; TCDD 100 ng/kg/day IP injection in DMSO, GD 8–14 [54].

## Data Availability

No new data were generated or analysed in support of this research.
